# IgG4-related pleural effusion with high adenosine deaminase levels: A case report and literature review

**DOI:** 10.1097/MD.0000000000025162

**Published:** 2021-03-19

**Authors:** Masafumi Shimoda, Yoshiaki Tanaka, Kozo Morimoto, Masao Okumura, Kiyomi Shimoda, Tamiko Takemura, Teruaki Oka, Takashi Yoshiyama, Kozo Yoshimori, Ken Ohta

**Affiliations:** a Respiratory Disease Center; b Department of Thoracic Surgery, Fukujuji Hospital, Japan Anti-tuberculosis Association, Kiyose City, Tokyo; c Kanagawa Cardiovascular and Respiratory Center, Department of Pathology, Tomioka-higashi, Kanazawa-ku, Yokohama, Kanagawa; d Department of Pathology, Fukujuji Hospital, Japan Anti-tuberculosis Association, Kiyose City, Tokyo, Japan.

**Keywords:** adenosine deaminase, IgG4-related disease, pleural effusion, tuberculous pleurisy

## Abstract

**Rationale:**

Levels of pleural fluid adenosine deaminase (ADA), a useful marker for the diagnosis of tuberculous pleurisy, are elevated in some reports of immunoglobulin G4 (IgG4)-related pleural effusion. We describe a patient with IgG4-related pleural effusion who exhibited a high concentration of ADA. Furthermore, we reviewed the literature to compare patients with IgG4-related pleural effusion and tuberculous pleurisy.

**Patient concerns:**

A 75-year-old male patient had dyspnea for 1 month with a left pleural effusion that was exudative, lymphocyte dominant. The pleural fluid test results revealed a total protein (TP) concentration of 6.60 g/dl, a lactate dehydrogenase (LDH) level of 383 IU/dl, and an ADA concentration of 54.5 U/L. An interferon gamma release assay showed a negative result.

**Diagnoses:**

Histological analysis of the thoracoscopic pleural biopsy revealed lymphoplasmacytic infiltration, with 80 IgG4-positive plasma cells/high-power field, and an IgG4/IgG ratio of approximately 40% to 50%. Other diseases were ruled out based on symptoms, negative autoimmune antigen results, and histopathologic findings. Thus, he was diagnosed with IgG4-related pleural effusion.

**Interventions:**

He received 15 mg of prednisolone as therapy.

**Outcomes:**

His pleural effusion and symptoms improved gradually within several months, and prednisolone was tapered to 6 mg daily.

**Lessons:**

It is important to distinguish between IgG4-related pleural effusion and tuberculous pleurisy. Therefore, we compared 22 patients with IgG4-related pleural effusion from PubMed and the Japan Medical Abstracts Society to 40 patients with tuberculous pleurisy at Fukujuji Hospital from January 2017 to May 2019. According to thoracentesis findings, 14 of 18 patients with IgG4-related pleural effusion had high ADA more than 40 U/L. The pleural effusion of patients with IgG4-related pleural effusion showed higher TP levels (*P* < .001) and lower LDH (*P* < .001) and ADA levels (*P* = .002) than those with tuberculous pleurisy. Moreover, the pleural fluid ADA/TP ratio was a good predictor for differentiating IgG4-related pleural effusion and tuberculous pleurisy (area under the receiver operating characteristic curve of 0.909; 95% confidence level: 0.824–0.994).

## 1 Introduction

Immunoglobulin G4 (IgG4)-related pleural effusion is a rare phenotype of IgG4-related disease (IgG4-RD),^[1]^ which is a chronic and systemic fibroinflammatory disorder that includes Mikulicz disease, autoimmune pancreatitis, Riedel thyroiditis, interstitial nephritis, and retroperitoneal fibrosis.^[1]^ Histopathologic findings of IgG4-RD involve the appearance of dense lymphoplasmacytic infiltrates with abundant IgG4-positive plasma cells and characteristic fibrosis.^[1,2]^ The rates of IgG4-related pleural effusion are reportedly 1.6% among patients with IgG4-RD and 4.6% among IgG4-RD patients with intrathoracic lesions.^[3]^ In general, IgG4-related pleural effusion is exudative with a predominance of lymphocytes and/or plasma cells,^[2]^ though these clinical features are not fully understood.^[4]^ Some reports of IgG4-related pleural effusion have indicated an adenosine deaminase (ADA) level higher than 40 U/ml in pleural effusion.^[5–7]^ The level of ADA in pleural fluid is generally used as a marker for the diagnosis of tuberculous pleurisy^[8–10]^ and it is therefore difficult to differentiate between IgG4-related pleural effusion and tuberculous pleurisy.^[5–7]^

Here, we describe a patient with IgG4-related pleural effusion who had a high concentration of ADA. Furthermore, we compare IgG4-related pleural effusion with tuberculous pleurisy based on a literature review.

## 2 Case presentation

This case involves a 75-year-old male patient who had dyspnea for 1 month prior to admission. He had a medical history of chronic obstructive pulmonary disease, diabetes mellitus, hepatitis B, and gastric polyps. He had a smoking history (45 pack-years) and was allergic to mackerel. He was diagnosed with autoimmune pancreatitis by his previous doctor and began 5.0 mg of oral prednisolone daily when he was 67 years old; his prednisolone dosage was decreased to 2.5 mg daily one year ago. He visited our hospital 8 months prior due to cough and sputum. He was diagnosed with asthma and chronic obstructive pulmonary disease overlap because of obstructive ventilatory disturbance and reversibility by short-acting β2 agonists by spirometry. His symptoms had been improved by treatment with inhaled vilanterol trifenatate/fluticasone furoate and oral montelukast sodium. Nonetheless, dyspnea gradually developed, as did a painless left pleural effusion on chest radiography, and he was thus admitted to our hospital.

**Figure 1:**
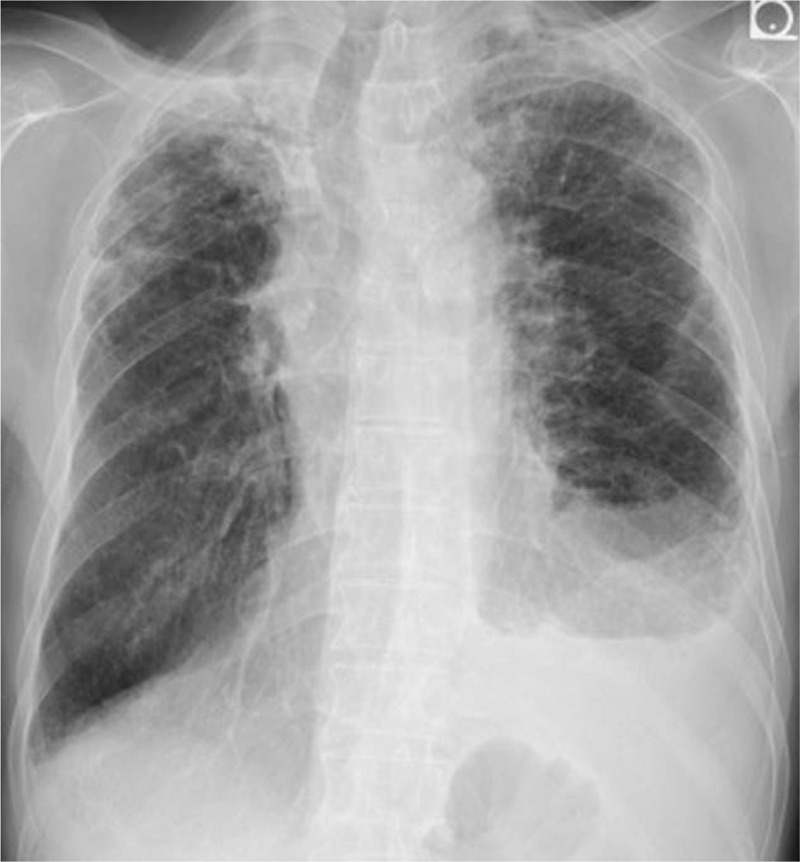
A chest radiograph showing left pleural effusion.

**Figure 2:**
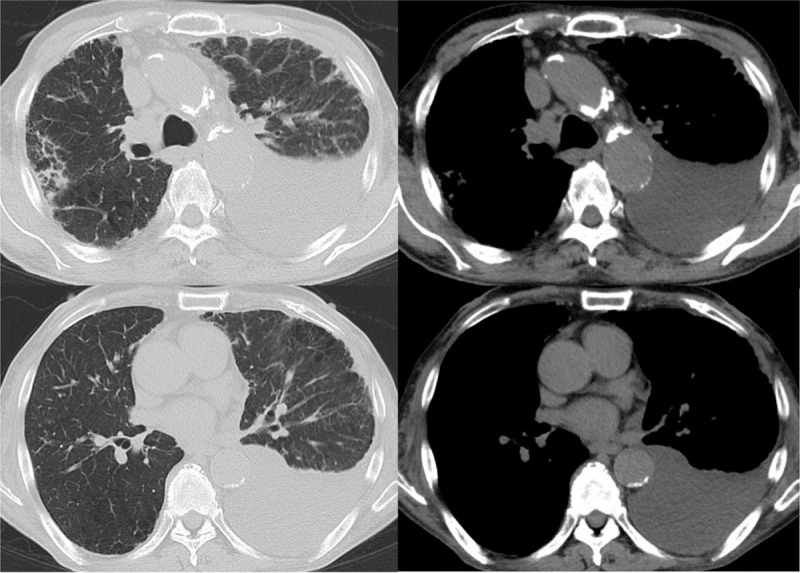
A chest high-resolution computed tomography scan. Chest CT showed a moderate amount of left pleural effusion, peripheral consolidation with interlobular septal thickening in mainly the bilateral upper lobe, emphysema, and swelling of the mediastinal lymph nodes.

His vital signs were normal, and physical examinations revealed no abnormalities. Laboratory findings were as follows: white blood cell count 11,220/μl, with 76.5% polymorphic nuclear leukocytes and 5.5% eosinophils; C-reactive protein 0.29 mg/dl; hemoglobin A1c 6.5%; serum IgG level 3556 mg/dl, including 1310 mg/dl of IgG4; and serum immunoglobulin E level 3968 U/ml (Table 1). An interferon gamma release assay showed a negative result (T-SOPT; panel A-nil 0 spot, panel B-nail -1 spot, positive control 551 spots, and negative control 2 spots). Chest radiography and a chest computed tomography (CT) scan showed a moderate amount of left pleural effusion (Figs. 1 and 2). Additionally, CT revealed peripheral consolidation with interlobular septal thickening in mainly the bilateral upper lobe, emphysema, and swelling of the mediastinal lymph nodes. ^18^F-fluorodeoxyglucose positron emission tomography imaging (FDG-PET/CT) showed accumulation of fluorodeoxyglucose in the left pleura and mediastinal lymph nodes as well as peripheral consolidation in the right upper lobe and prostate gland. No abnormal pancreatic findings were detected by CT scan or FDG-PET/CT. The pleural effusion obtained by thoracentesis showed a total cell count of 4907/μl (lymphocytes 83.5%), total protein (TP) of 6.60 g/dl, lactate dehydrogenase (LDH) of 383 IU/dl, and ADA of 54.5 U/L. Pleural fluid bacterial culture, tuberculosis examination (smear, culture, and polymerase chain reaction tests), and cytology were negative.

**Table 1:** Laboratory findings for the patient.

Peripheral blood	
Hemoglobin (N: 14–18)	16.2 g/dl
Hematocrit (N: 40–48)	49.8%
RBC (N: 410 × 10^4^−530 × 10^4^)	565 × 10^4^/μl
WBC (N: 4000–8000)	11220/μl
Neutrophils (N: 48–61)	76.5%
Eosinophils (N: 1–5)	5.5%
Basophils (N: 0–1)	0.7%
Monocytes (N: 4–7)	6.8%
Lymphocytes (N: 25–45)	10.5%
Platelet (N: 13 × 10^4^−35 × 10^4^)	29.0 × 10^4^/μl
Coagulation	
PT-INR (N: 0.6–1.5)	1.16
APTT (N: 24–42)	29.0 sec
Fibrinogen (N: 200–400)	342 mg/ml
FDP (N: < 5.0)	9.3 μg/ml
D-dimer (N: < 1.0)	5.7 μg/ml
Serology	
C-reactive protein (N: 0–0.3)	0.29 mg/dl
IgG (N: 870–1700)	3556 mg/dl
IgG4 (N: 4.5–117)	1310 mg/dl
IgA (N: 110–410)	320 mg/dl
IgM (N: 33–190)	80 mg/dl
IgE (N: 0–400)	3968 IU/ml
KL-6 (N: 0–500)	273 U/ml
anti-CCP	negative
Antinuclear antibody	negative
SS-A	negative
IGRA	negative
Blood biochemistry	
Na (N: 135–145)	138 mmol/L
K (N: 3.5–5)	3.9 mmol/L
Cl (N: 98–108)	102 mmol/L
Blood urea nitrogen (N: 8–20)	22.0 mg/dl
Creatinine (N: 0.53–1.00)	0.77 mg/dl
Total protein (N: 6.7–8.3)	8.51 g/dl
Albumin (N: 3.8–5.1)	3.05 g/dl
Total bilirubin (N: 0.2–1.2)	0.9 mg/dl
AST (N: 115–359)	25 IU/L
ALT (N: 8–42)	19 IU/L
LDH (N: 119–229)	206 IU/L
Creatinine kinase (N: 62–287)	91 IU/L
HbA1c (N: 4.6–6.2)	7.7%
NT-pro BNP (N: < 125)	62.3 pg/ml
Tumor markers	
CEA (N: < 5.0)	0.5 ng/ml
CYFRA (N: < 3.5)	0.6 ng/ml
SCC (N: < 1.5)	1.0 ng/ml
ProGRP (N: < 81)	55.4 pg/ml
sIL-2 receptor (N: 122–496)	945 U/ml
Thoracentesis findings	
Total cell count	4907/μl
Neutrophils	4.0%
Lymphocytes	83.5%
Eosinophils	8.0%
Macrophages	2.5%
Total protein	6.60 g/dl
Lactate dehydrogenase	383 IU/L
Glucose	101 mg/dl
Adenosine deaminase	54.5 U/L
IgG	3979 U/L
IgG4	1510 U/L

ALT = alanine transaminase, anti-CCP = anti-cyclic citrullinated peptide antibody, APTT = activated partial thromboplastin time, AST = aspartate aminotransferase, CEA = carcinoembryonic antigen, CI = chloride, CYFRA = cytokeratin 19 fragment, FDP = fibrin/fibrinogen degradation products, HbA1c = hemoglobin A1c, Ig = immunoglobulin, IGRA = interferon gamma release assay, K = potassium, KL-6 = Krebs von den Lungen-6, LDH = lactate dehydrogenase, N = normal range, Na = sodium, NT-pro BNP = N-terminal probrain natriuretic peptide, proGRP = progastrin-releasing peptide, PT-INR = International Normalized Ratio, RBC = red blood cells, SCC = squamous cell carcinoma antigen, sIL-2R = soluble interleukin-2 receptor, SS-A = anti-Sjögren's syndrome-related antigen A, WBC = white blood cells.

**Figure 3:**
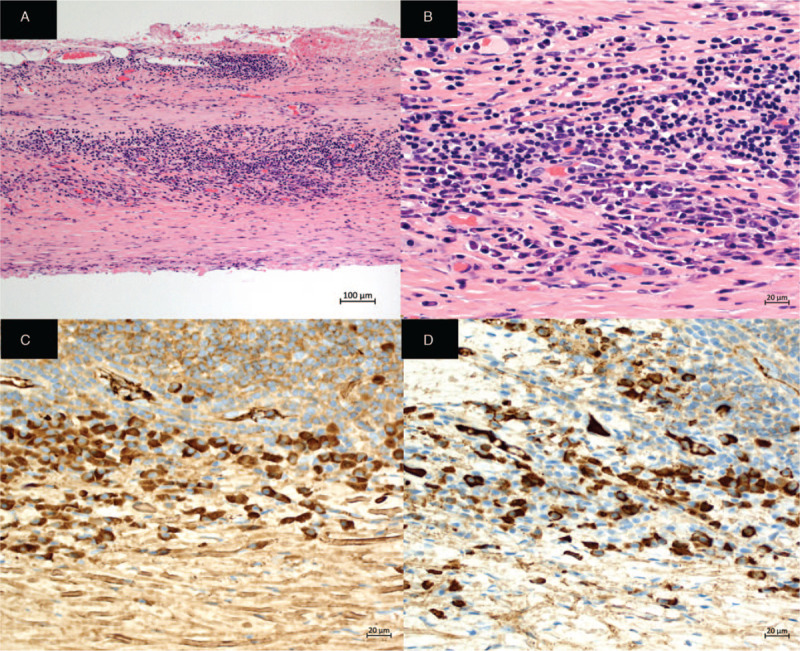
Histological findings of the thoracoscopic left pleural biopsy. The left parietal pleural biopsy specimens revealed the presence of dense collagenous fibrosis with lymphoplasmacytic and plasma cell infiltration without storiform fibrosis and obstructive phlebitis. It did not show malignancy, granuloma, or lymphoid follicles. Based on immunohistochemical staining, 80 IgG4-positive plasma cells/high-power field and an IgG4/IgG ratio of approximately 40% to 50% were obtained. A: Hematoxylin–eosin (H&E) staining × 100, B: H&E staining × 200 C: Immunohistochemical staining for IgG × 400, D: Immunohistochemical staining for IgG4 × 400.

Because of the high concentrations of ADA in the pleural effusion, we could not distinguish between IgG4-related pleural effusion and tuberculous pleurisy. Therefore, we performed a thoracoscopic pleural biopsy, and histological analysis revealed the presence of dense collagenous fibrosis with lymphoplasmacytic infiltration with no malignancy or granuloma (Fig. 3). Immunohistochemical staining showed 80 IgG4-positive plasma cells/high-power field and an IgG4/IgG ratio of approximately 40% to 50%. Hence, he was diagnosed with IgG4-related pleural effusion since the findings fulfilled the diagnostic criteria for IgG4-RD.^[11]^ Treatment with prednisolone 15 mg daily orally was started, and his pleural effusion and symptoms improved gradually within several months. Moreover, the mediastinal lymph nodes and interstitial changes in his lung also improved according to chest radiography. Prednisolone was tapered to 6 mg daily, and he has since remained well with no recurrence of symptoms during over the one-year follow-up period. (data availability statement).

## 3 Discussion

We report a case of IgG4-related pleural effusion with a high concentration of ADA in the pleural effusion. In this case, it was difficult to distinguish IgG4-related pleural effusion from tuberculous pleurisy without performing thoracoscopic pleural biopsy. The pleural fluid IgG4 level was elevated, similar to the serum IgG4 level. His mediastinal lymph nodes, lung, and prostate gland might be also affected by IgG4-RD due to observed accumulation by FDG-PET/CT. However, histopathological examinations of those organs were not performed. Chest radiography showed that the interstitial changes and mediastinal lymph nodes in his lung improved after starting steroid therapy.

The most instructive point in our report is the high concentration of pleural fluid ADA, even though it was not due to tuberculous pleurisy. ADA is an enzyme participating in purine catabolism in the pathway from adenosine to inosine and is produced by lymphocytes.^[12,13]^ The level of pleural fluid ADA is a useful marker for the diagnosis of tuberculous pleurisy.^[8–10]^ Conversely, pleural fluid smear microscopy and cultures show low rates of positivity for the diagnosis of tuberculous pleurisy (as low as 6% and 36%, respectively).^[14]^ Even polymerase chain reaction tests (sensitivity 51.4%) and interferon gamma release assay (sensitivity 72%) for pleural effusion have suboptimal diagnostic accuracies with regard to tuberculous pleurisy.^[15,16]^ The most widely accepted cut-off value of pleural fluid ADA for diagnosing tuberculous pleurisy is 40 U/L, with a sensitivity and specificity of 92% and 90%, respectively.^[17]^ Therefore, an elevated pleural fluid ADA level is useful for diagnosing tuberculous pleurisy.^[8–10,17]^ Diseases with high pleural fluid ADA levels other than tuberculosis include empyema, malignancies, infectious diseases, and connective tissue diseases such as rheumatoid arthritis.^[8,9]^ Castro et al reported that 1.71% of nontuberculous lymphocytic pleural effusions show a high ADA level, exceeding 40 U/L.^[18]^ The mechanism of ADA production suggests that the interaction of T cells with B cells may induce the activation of cell-mediated immunity,^[5]^ but it has not been fully elucidated.^[4,9]^ ADA is categorized as ADA-1 and ADA-2, and ADA-2, which is induced by the stimulation of monocytes/macrophages, is mainly activated in tuberculous pleural effusion.^[9,19]^ Low ADA-2 activities are observed among other diseases, such as neoplastic exudate, empyema, and parapneumonic exudate.^[9,19]^ However, only total ADA can be routinely measured at our clinical practice. Regarding IgG4-related pleural effusion, a previous review of 8 cases showed slightly elevated pleural fluid ADA levels, with a median of 32.2 U/L (range 26.6–50.1), which was significantly higher than that in non-IgG4-related cases without tuberculous pleurisy (*P* = .05).^[4]^ Considering that ADA levels are elevated in both IgG4-related pleural effusion and tuberculous pleurisy, it is very important to distinguish between them because of the different treatments. Indeed, the first-line treatment for IgG4-RD is glucocorticoids,^[2]^ but glucocorticoids influence the development of tuberculous infection.^[20]^ Therefore, the comparison between IgG4-related pleural effusion and tuberculous pleurisy is very important.

In addition to the current case, we collected past case reports of IgG4-related pleural effusion from PubMed and the Japan Medical Abstracts Society database using the keywords “IgG4 pleuritis,” “IgG4 pleural effusion,” and “Mikulicz's disease pleural effusion”. The search was limited to in English and Japanese languages only. We searched using those keywords in Japanese in the Japan Medical Abstracts Society database and excluded reports without an English abstract. Furthermore, we retrospectively studied patients who were diagnosed with tuberculous pleurisy due to tubercle bacilli cultured from pleural effusion at the Respiratory Disease Center of Fukujuji Hospital from January 2017 to March 2019. We excluded the cases with no laboratory data. Among cases with suspected IgG4-related pleural effusion, we also excluded those that did not meet the diagnostic criteria for IgG4-related disease.^[11]^ All data were analyzed and processed using EZR, version 1.35.^[21]^

**Figure 4:**
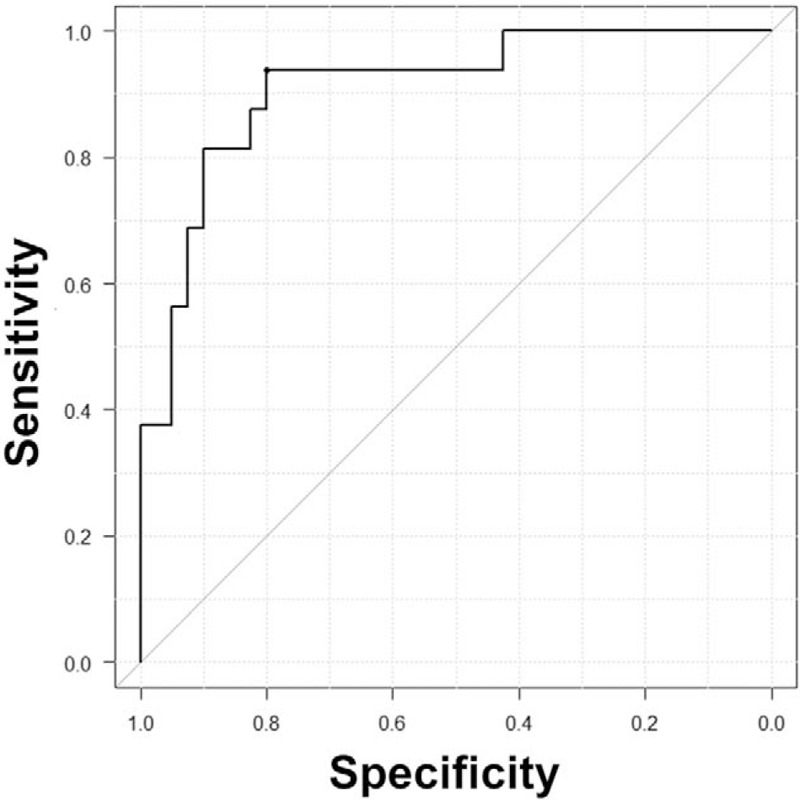
The area under the receiver operating characteristic curve of the pleural fluid ADA/TP ratio for differentiating between IgG4-related pleural effusion and tuberculous pleurisy. The area under the curve was 0.909 (95% CI: 0.824–0.994). The predictive accuracy at the cut-off value was less than 15 points, with a sensitivity of 93.8%, specificity of 72.5%, and odds ratio of 36.8 (95% CI: 4.7–1702.4). CI = confidence interval.

We collected 22 patients with IgG4-related pleural effusion, including our patient,^[5–7,22–39]^ and 40 patients with tuberculous pleurisy. Table 2 shows the characteristics of the patients. Age, sex, and underlying disease were not significantly different. Overall, the median white blood cell count and C-reactive protein levels of IgG4-related pleural effusion were in the normal range, which is similar to a past report.^[4]^ Regarding thoracentesis findings, lymphocytes were dominant in both diseases, with no significant difference (*P* = .629). The median pleural fluid ADA level in IgG4-related pleural effusion was elevated to 55.7 U/L (interquartile range (IQR) 42.3–71.6), which was lower than that in tuberculous pleurisy (median (IQR): 83.9 U/L (60.4–109.4), *P* = .002). Of 18 patients with IgG4-related pleural effusion, 14 (77.8%) showed a high pleural fluid ADA of more than 40 U/L. Patients with IgG4-related pleural effusion showed higher pleural fluid TP [median (IQR): 5.75 g/dl (4.89–6.23) vs 4.65 g/dl (3.77–5.13), *P* < .001] and pleural fluid glucose [median (IQR): 122 mg/dl (77–116) vs 65.5 mg/dl (51.3–94.3), *P* = .012], and lower pleural fluid LDH [median (IQR): 167 IU/L (137–290) vs 514 IU/L (293–1180), *P* < .001] than those with tuberculous pleurisy. The pleural fluid ADA/TP ratio is a good predictor for differentiating between IgG4-related pleural effusion and tuberculous pleurisy, with an area under the receiver operating characteristic curve of 0.909 (95% confidence interval (CI): 0.824–0.994) (Fig. 4). When the cut-off value was less than 15 points as decided by a point of maximum sensitivity and specificity, the sensitivity and specificity were 93.8% and 72.5%, respectively, and the odds ratio was 36.8 (95% CI: 4.7–1702.4). Pleural fluid LDH was a good predictor [area under the receiver operating characteristic curve 0.819 (95% CI: 0.702–0.936)]. A previous study reported that the pleural fluid LDH levels in patients with tuberculous pleurisy differed between positive and negative pleural effusion cultures (positive culture vs negative culture; median 680 IU/L [IQR: 455–1055) vs median 550 IU/L (IQR: 389–763), *P* < .001].^[40]^ All tuberculosis patients in our study showed positive pleural fluid culture results; thus, we could not conclude that the pleural fluid LDH level was also useful for distinguishing between patients with IgG4-related pleural effusion and tuberculous pleurisy with negative pleural effusion cultures. Therefore, we adapted TP and ADA as prediction criteria. Among 14 patients with IgG4-related pleural effusion who were investigated for pleural fluid IgG4, the median pleural fluid IgG4 level was 1120 mg/dl (IQR 571–1520), which was slightly higher than the serum IgG4 level (pleural fluid/serum IgG4 ratio; median (IQR) 1.12 (0.76–1.27)). A high concentration of pleural fluid IgG4 might be useful for diagnosing IgG4-related pleural effusion; however, we were unable to compare this for patients with tuberculous pleurisy because of a lack of data. In 9 of 22 patients with IgG4-related pleural effusion, organs other than pleuritis were not affected.

**Table 2:** Baseline characteristics of the patients in the literature review.

	IgG4-related disease (n = 22)	Tuberculous pleurisy (n = 40)	*P* value
Age, median (IQR), years	70.0 (65.0–76.0)	67.0 (43.5–78.5)	.515
Sex (male/female)	20/2	32/8	.472
Comorbidity, n (%)^∗^	19 (90.5)	28 (73.7)	.182
Laboratory findings			
WBC, median (IQR), cells/μl^†^	5780 (4750–6225)	6780 (5350–8615)	.088
CRP, median (IQR), mg/dl^†^	0.29 (0.14–1.25)	8.21 (4.30–13.1)	<.001
LDH, median (IQR), U/L^§^	174 (167–188)	189 (165–233)	.146
Total protein, median (IQR), g/dl^||^	7.75 (6.93–8.63)	6.56 (5.61–7.07)	.005
Albumin, median (IQR), g/dl^§^	2.90 (2.40–3.18)	2.51 (1.79–3.12)	.219
Radiological findings			
Right pleural fluid, n (%)	9 (40.9)	24 (60.0)	.188
Left pleural fluid, n (%)	6 (27.3)	10 (25.0)	1.000
Bilateral pleural fluid, n (%)	7 (31.8)	6 (15.0)	.191
Thoracentesis findings			
Neutrophils, median (IQR), %^¶^	4.0 (1.0–11.0)	10.0 (4.8–39.5)	.061
Lymphocytes, median (IQR), %^#^	81.4 (69.5–89.6)	82.0 (56.3–89.8)	.579
LDH, median (IQR), IU/L^∗∗^	176 (137–290)	514 (293–1180)	<.001
Total protein, median (IQR), g/dl^††^	5.75 (4.89–6.23)	4.65 (3.77–5.13)	<.001
Glucose, median (IQR), mg/dl^‡‡^	112 (77–116)	65.5 (51.3–94.3)	.012
ADA, median (IQR), U/L^§§^	55.7 (42.3–71.6)	83.9 (60.4–109.4)	.002

∗ IgG4 n = 21, TB n = 38.

† IgG4 n = 16, TB n = 39.

† IgG4 n = 17, TB n = 39.

§ IgG4 n = 11, TB n = 39.

|| IgG4 n = 12, TB n = 38.

¶ IgG4 n = 13, TB n = 39.

# IgG4 n = 14, TB n = 39.

∗∗ IgG4 n = 17, TB n = 40.

†† IgG4 n = 20, TB n = 40.

‡‡ IgG4 n = 13, TB n = 40.

§§ IgG4 n = 18, TB n = 40. ADA = adenosine deaminase, CRP = C-reactive protein, IQR = interquartile range, LDH = lactate dehydrogenase, Statistics method = Student *t* test, Mann–Whitney U test, and Fisher exact test, WBC = white blood cell count.

This study had several limitations. The review was conducted retrospectively in a single-center hospital, and some medical data were not recorded. In addition, publication bias exists because the data for many of the patients with IgG4-related pleural effusion were obtained from published reports.

## 4 Conclusion

We report a patient with IgG4-related pleural effusion, which is difficult to distinguish from tuberculous pleurisy due to a high pleural fluid ADA level. The pleural fluid ADA/TP ratio is useful for distinguishing IgG4-related pleural effusion from tuberculous pleurisy.

## Author contributions

**Conceptualization:** Masafumi Shimoda, Yoshiaki Tanaka.

**Data curation:** Masafumi Shimoda, Yoshiaki Tanaka, Kozo Morimoto, Masao Okumura, Kiyomi Shimoda, Tamiko Takemura, Teruaki Oka, Takashi Yoshiyama, Kozo Yoshimori.

**Formal analysis:** Masafumi Shimoda.

**Investigation:** Masafumi Shimoda, Kiyomi Shimoda, Tamiko Takemura, Teruaki Oka.

**Methodology:** Masafumi Shimoda, Yoshiaki Tanaka.

**Project administration:** Masafumi Shimoda, Kozo Yoshimori, Ken Ohta.

**Resources:** Masafumi Shimoda.

**Software:** Masafumi Shimoda.

**Supervision:** Yoshiaki Tanaka, Kozo Morimoto, Takashi Yoshiyama, Kozo Yoshimori, Ken Ohta.

**Validation:** Masafumi Shimoda, Yoshiaki Tanaka.

**Visualization:** Masafumi Shimoda.

**Writing – original draft:** Masafumi Shimoda.

**Writing – review & editing:** Masafumi Shimoda, Yoshiaki Tanaka.

## Supplementary Material

Supplemental Digital Content
